# Discrimination based on criminal record and healthcare utilization among men recently released from prison: a descriptive study

**DOI:** 10.1186/2194-7899-2-6

**Published:** 2014-03-25

**Authors:** Joseph W Frank, Emily A Wang, Marcella Nunez-Smith, Hedwig Lee, Megan Comfort

**Affiliations:** 1 Division of General Internal Medicine, University of Colorado School of Medicine, Aurora, CO US; 2 Denver Veterans Affairs Medical Center, Denver, CO USA; 3 Section of General Internal Medicine, Yale University School of Medicine, New Haven, CT USA; 4 Department of Sociology, University of Washington, Seattle, WA USA; 5 Department of Medicine, University of California San Francisco, San Francisco, CA USA; 6 Urban Health Program, RTI International, San Francisco, CA USA

## Abstract

**Background:**

Healthcare discrimination based on race/ethnicity is associated with decreased healthcare access and utilization among racial/ethnic minority patients. Discrimination based on criminal record may also negatively impact healthcare access and utilization among ex-prisoners.

**Methods:**

We conducted a secondary analysis of data from a cross-sectional survey of 172 men recently released from state prison. We examined the association between self-reported criminal record discrimination by healthcare workers and utilization of 1) emergency department (ED) and 2) primary care services. We created staged logistic regression models, adjusting for sociodemographic characteristics and self-reported racial/ethnic discrimination.

**Results:**

Among 172 male participants, 42% reported a history of criminal record discrimination by healthcare workers. Participants who reported discrimination were older (mean, 42 vs. 39 years; p = .01), more likely to be college educated (26% vs. 11%; p = .03), and had more extensive incarceration histories (median years incarcerated, 16 vs. 9; p = .002) compared to those who did not report discrimination. Self-reported criminal record discrimination by healthcare workers was significantly associated with frequent ED utilization [odds ratio (OR) = 2.7, 95% confidence interval 24 (CI) 1.2-6.2] but not infrequent primary care utilization [OR = 1.6, 95% CI 0.7-3.8].

**Conclusions:**

Recently released prisoners report criminal record discrimination by healthcare workers, and this experience may impact healthcare utilization. Future studies should seek to further characterize criminal record discrimination by healthcare workers and prospectively examine its impact on health outcomes.

**Electronic supplementary material:**

The online version of this article (doi:10.1186/2194-7899-2-6) contains supplementary material, which is available to authorized users.

## Background

In 2010, more than 2.3 million United States residents filled the nation’s prisons and jails, and another 4.9 million were under some form of correctional supervision in the community (Glaze & Bonczar 2011; Guerino et al. 2011; Minton 2011). In this large and growing population, prevalence of chronic diseases such as hypertension, asthma, hepatitis and HIV is high compared to the general population (Binswanger et al. 2009; Maruschak & Beavers 2009; Wilper et al. 2009). Yet prisoners may receive suboptimal care while incarcerated (Newman & Scott 2011) and former prisoners may have limited access to healthcare and an increased risk of mortality in the community (I. A. (Binswanger et al. 2007; Newman & Scott 2011; Kulkarni et al. 2010).

A possible contributor to poor health outcomes among people released from prison is discrimination. Prisoners and former prisoners are disproportionately racial and ethnic minorities and likely experience discrimination based on their racial or ethnic identity in healthcare settings. (Pew Center on the States 2009) The link between self-reported racial/ethnic discrimination and poor health and healthcare access is well documented (Paradies 2006; Shavers et al. 2012; Williams & Mohammed 2009). Self-reported racial/ethnic discrimination is associated with a lower likelihood of having a routine physical within the past year, delays in care, poor adherence to recommended care and decreased use of preventive services (Benjamins 2012; Blanchard & Lurie 2004; Casagrande et al. 2007; Hausmann et al. 2008; Trivedi & Ayanian 2006; Van Houtven et al. 2005). Possible mechanisms include medical mistrust, poor patient-provider communication and decreased satisfaction with care (Hausmann et al. 2011; LaVeist et al. 2000).

Discrimination based on one’s criminal record may serve as an additional barrier to engaging in healthcare (Schnittker & John 2007; Smedley et al. 2003). A criminal record has been identified as a stigmatized social status that can result in unfair treatment and daily indignities across a variety of social settings (Schnittker & John 2007; Uggen & Manza 2002). Discrimination based on criminal record has been firmly established in employment, housing, and receipt of other social services not only via self-reports from former prisoners, but also through the use of multiple audit studies and other novel experimental designs (Pager & Shepherd 2008; Pager et al. 2009b; Uggen et al. 2004). Given this body of evidence, it is plausible that patients with a history of incarceration also anticipate and perceive discrimination in the healthcare settings.

Unlike a patient’s race or ethnicity, a patient’s criminal record is not always readily apparent but can be revealed to healthcare providers in several ways. When receiving care in correctional healthcare settings, prisoners may question the loyalties of healthcare providers who work within the criminal justice system (Benatar & Upshur 2008). Also, when incarcerated patients are transferred to a community medical facility for evaluation, these patients are readily recognizable, clad in bright orange jumpsuits, shackles and with armed escort. A history of incarceration may be documented in a patient’s medical record or shared directly with a health professional, potentially leading to discussion of other commonly stigmatized topics such as mental illness, substance abuse or HIV risk behaviors (Marlow et al. 2010).

The aims of this study were to describe self-reported criminal record discrimination by healthcare workers, to describe its relationship with racial/ethnic discrimination and to examine the association between criminal record discrimination and healthcare utilization among recently released prisoners. As with racial/ethnic discrimination, the experience of criminal record discrimination by healthcare workers may foster patients’ mistrust, negatively impacting future relationships and communication with healthcare personnel. Such relationships are an essential feature of primary care compared to more episodic, relatively anonymous care in emergency department (ED) settings. Therefore, we hypothesized that the experience of criminal record discrimination by healthcare workers is associated with decreased utilization of primary care and increased utilization of ED services.

## Methods

### Study overview

We used data from the Relate Project ("HIV Risk Among Male Parolees and Their Female Partners"; R01 MH0787443), a cross-sectional survey of 172 male-female couples (N = 344) recruited based on release of the male partner from state prison in the previous 12 months.

### Recruitment and eligibility

Participants were recruited using street outreach methods, posting of flyers and presentations in community locations. Recruitment took place in Oakland and San Francisco, California. Eligibility criteria included both parties being 18 years of age or older, speaking English, being in a relationship with each other during the male partner’s most recent incarceration and remaining in a relationship at the time of eligibility screening, and the male partner being able to provide documentation of release from prison at least 3 and no more than 12 months prior to eligibility screening. As exposure to incarceration differed markedly by gender, we limited this analysis to male survey participants (N = 172).

### Data collection

Data were collected between January 2009 and February 2011. Participants were consented and interviewed in private rooms at community-based organizations. Interviews were administered using a combination of computer-assisted personal interviewing and audio computer-assisted self-interviewing. Participants were remunerated $50 each for their time. All study procedures were reviewed and approved by the University of California-San Francisco Committee on Human Research and the Research Triangle Institute International Institutional Review Board.

### Independent variable

The survey included questions modeled on the *General Ethnic Discrimination Scale*, which was adapted from the *Schedule of Racist Events* and validated in a multi-ethnic population comprised of both community-dwelling adults and college students (Klonoff & Landrine 1999; Landrine et al. 2006). In this survey, interviewers asked participants a series of 15 questions about discrimination based on criminal record. We defined self-reported lifetime history of criminal record discrimination by healthcare workers as an affirmative response to the following question: "How often have you been treated unfairly by people in helping jobs (like doctors, nurses, psychiatrists, case workers, dentists, school counselors, or social workers) because of your criminal record?" Participants who responded "Once in awhile, Sometimes, A lot, Most of the time and All of the time" were categorized as having experienced criminal record discrimination by healthcare workers.

### Dependent variables

We created two dependent variables measuring healthcare utilization in two different settings. To assess utilization of emergency care, we created a dichotomous variable capturing ED utilization based on responses to the question, "Since you turned 18 years old, how many times total have you been to the emergency room for your own medical condition?" We defined frequent ED utilization as the quartile of individuals with the most frequent reported ED utilization, (Six or more ED visits vs. five or fewer ED visits). To assess utilization of primary care, we created a dichotomous variable based on responses to the question, "What is the best description of how often you see a doctor or a nurse for a general check-up?" Response options included: Never, Less than once every five years, Once every five years, Once every two to four years, Once every two years, Once every year and More than once every year. We dichotomized responses to identify the quartile of participants reporting the least frequent utilization of primary care services (Once every 5 years or less vs. More than once every 5 years).

### Covariates of interest

We assessed sociodemographic variables including age (continuous), race/ethnicity (Black/White/other race), insurance status (insured/uninsured), employment status ("working steady job in the legal economy") and education level (less than high school education/high school diploma or GED/post-secondary education). We created a dichotomous indicator of the presence of any one of several self-reported medical diagnoses (asthma, diabetes, hypertension, epilepsy, sickle cell anemia, heart disease, obesity, HIV, hepatitis C, arthritis, cancer, tuberculosis, emphysema, sexually transmitted infection or stomach or digestion problems) as well as an indicator of the presence of any self-reported psychiatric diagnosis (anxiety, depression or post-traumatic stress disorder). Additionally, we created dichotomous variables indicating current tobacco use and current use of any of the following substances: marijuana, cocaine, stimulants, heroin, sedatives or painkillers not prescribed by a physician. We created a variable for each individual indicating the total number of lifetime incarcerations (in juvenile detention facilities, jails and state or federal prisons) and total time incarcerated in years. We created a dichotomous variable capturing any contact with correctional healthcare based on responses to the question, "The last time you were incarcerated, how many times did you see a doctor or a nurse when you requested to see one?" Finally, similar to our independent variable, we defined self-reported lifetime history of racial/ethnic discrimination by healthcare workers as any affirmative response to the following question: "How often have you been treated unfairly by people in helping jobs (like doctors, nurses, psychiatrists, case workers, dentists, school counselors, or social workers) because of your race/ethnic group?"

### Statistical analysis

We used *t* tests or Wilcoxon rank-sum tests for bivariate comparisons of continuous data and chi-square tests for bivariate comparisons of proportions. We created three sets of logistic regression models to examine the association of self-reported criminal record discrimination by healthcare workers with each dependent variable. In the first set, we generated unadjusted odds ratios and 95% confidence intervals (Model 1). In the second set, we adjusted for age, race/ethnicity, insurance status, recent contact with correctional healthcare and total time incarcerated (Model 2). Total time incarcerated was logarithmically transformed to account for the nonlinear nature of the measure. We selected covariates based on *a priori* hypotheses of the characteristics likely to confound the association of interest. These hypotheses were guided by literature documenting differences in both discrimination and healthcare utilization according to age, race/ethnicity and socioeconomic status (Kessler et al. 1999; Wang et al. 2013). Additionally, we hypothesized that both total exposure to incarceration and recent contact with correctional healthcare are associated with both discrimination and healthcare utilization (Mellow & Greifinger 2007; Patterson 2013). Finally, in Model 3, we further adjusted for both self-reported racial/ethnic discrimination by healthcare workers as well as the interaction between both discrimination variables. We examined this interaction based on prior evidence of an additive effect of these stigmatized characteristics in other domains such as employment (Pager et al. 2009a). We performed all analyses using SAS version 9.3 (SAS Institute Inc, Cary, NC). All p values are 2-tailed. We considered p < 0.05 significant.

## Results

Overall, 42% of participants reported a lifetime history of criminal record discrimination by healthcare workers (Table 1). Among those reporting criminal record discrimination by healthcare workers, 68% also attributed racial/ethnic discrimination to healthcare workers (p < .001). Individuals reporting criminal record discrimination by healthcare workers were significantly older (mean age, 42.3 vs. 38.6, p = 0.01), more likely to have obtained post-secondary education (26% vs. 11%, p = 0.03) and had a more extensive incarceration history (median years incarcerated, 16 vs. 9, p = 0.002) compared with individuals reporting no criminal record discrimination. Criminal record discrimination varied across racial/ethnic groups as 56% of White participants reported discrimination based on criminal record compared to 41% of Black participants and 39% of those of other race/ethnicities, respectively (p = 0.48). Finally, individuals reporting criminal record discrimination by healthcare workers were more likely to report a psychiatric diagnosis (45% vs. 38%, p *=* 0.43) and to report a healthcare visit during their most recent incarceration (75% vs. 62%, p = 0.07) though these associations were not statistically significant.

**Table 1:** **Characteristics of total sample and sample stratified by self-reported lifetime healthcare discrimination based on criminal record**

	Total sample (N = 172)	Any reported discrimination (N = 73)	No reported discrimination (N = 99)
Age (years) (mean, SD)*	40.2 (9.0)	42.3 (8.3)	38.6 (9.3)
Race/ethnicity			
*Black*	73%	71%	75%
*White*	10%	14%	8%
*Other race*†	16%	15%	17%
Uninsured	49%	42%	54%
Unemployed	80%	79%	80%
Education*			
*Less than high school*	33%	33%	33%
*High school diploma or GED*	49%	41%	56%
*Postsecondary education*	17%	26%	11%
Medical history			
*Any medical diagnosis*‡	74%	77%	73%
*Any psychiatric diagnosis*§	41%	45%	38%
Substance use history			
*Current tobacco use*	74%	75%	74%
*Current drug use ║*	56%	58%	55%
Incarceration history			
Number of times incarcerated (median, IQR)*	24 (14–40)	28 (15–41)	20 (12–37)
Number of years incarcerated (median, IQR)*	11 (7–18)	16 (10–19)	9 (6–15)
*Healthcare visit during last incarceration*	67%	75%	62%
Any reported racial/ethnic discrimination*	44%	68%	25%

*SD = Standard deviation; IQR = Interquartile range.*

*p < .05.

^†^ *Includes Latino (N = 15), Native American (N = 7) and Other (N = 6).*

^‡^ *Includes any diagnosis of asthma, diabetes, hypertension, epilepsy, sickle cell anemia, heart disease, obesity, HIV, hepatitis C, arthritis, cancer, tuberculosis, emphysema, sexually transmitted infection or stomach or digestion problems.*

§*Include any diagnosis of anxiety, depression or post-traumatic stress disorder.*

║ *Includes current use of any of the following: marijuana, cocaine, stimulants, heroin, sedatives or painkillers not prescribed by a physician.*

**Figure 1:**
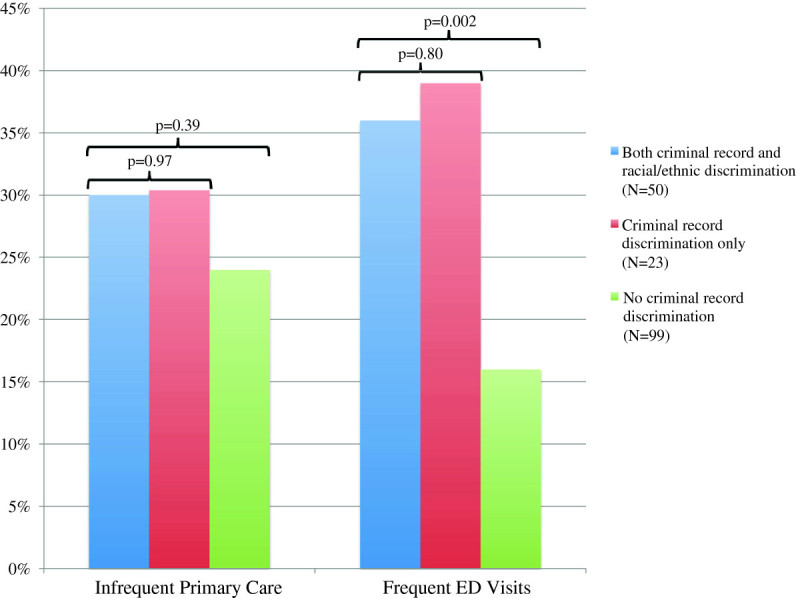
**Healthcare utilization stratified by self-reported criminal record and racial/ethnic discrimination by healthcare workers.** *ED = Emergency Department. P values represent pairwise comparisons using chi-square test. Groups are mutually exclusive.*

In unadjusted analyses, self-reported criminal record discrimination by healthcare workers was significantly associated with frequent ED utilization (37% vs. 16%%; p = .002) but not with infrequent primary care utilization (30% vs. 24%%; p = .39). Reported utilization did not differ among participants who also reported racial/ethnic discrimination by healthcare workers compared to those who reported criminal record discrimination only (Figure 1). Model 1 presents these unadjusted associations as crude odds ratios for criminal record discrimination (Table 2). In multivariable analyses adjusting for sociodemographic and correctional characteristics (Model 2), self-reported criminal record discrimination by healthcare workers remained significantly associated with frequent ED utilization (odds ratio [OR] 2.7, 95% confidence interval [CI] 1.2-5.8) but not infrequent primary care utilization (OR 2.1, 95% CI 0.9-4.5). In Model 3, the association between criminal record discrimination by healthcare workers and frequent ED utilization (OR 2.7, 95% CI 1.1-6.3) was not attenuated by adjustment for self-reported racial/ethnic discrimination and the interaction between the two forms of self-reported discrimination. Of note, racial/ethnic discrimination by healthcare workers was significantly associated with infrequent primary care utilization (OR 2.5, 95% CI 1.1-5.8) but not frequent ED utilization.

**Table 2:** **Multivariable logistic regression models estimating the association between self-reported criminal record discrimination by healthcare workers and healthcare utilization**

	Infrequent primary care utilization (N = 46)			Frequent ED utilization (N = 43)		
	Model 1	Model 2	Model 3	Model 1	Model 2	Model 3
	Crude OR (95% CI)	AOR (95% CI)	AOR (95% CI)	Crude OR (95% CI)	AOR (95% CI)	AOR (95% CI)
Criminal record discrimination	1.3 (0.7–2.7)	2.1 (0.9–4.5)	1.6 (0.7–3.8)	3.0 (1.5–6.2)*	2.7 (1.2–5.8)*	2.7 (1.2–6.2)*
Age (years)	---	1.0 (0.9–1.0)	1.0 (0.9–1.0)	---	1.1 (1.0–1.1)	1.1 (1.0–1.1)
Race/ethnicity						
*Black*	---	REF	REF	---	REF	REF
*White*	---	0.9 (0.3–3.2)	1.0 (0.3–3.4)	---	1.8 (0.6–5.3)	1.8 (0.6–5.4)
*Other race*	---	2.3 (0.9–5.6)	2.1 (0.8–5.3)	---	1.2 (0.4–3.5)	1.2 (0.4–3.4)
Uninsured	---	2.8 (1.3–5.9)*	2.9 (1.3–6.3)*	---	0.6 (0.3–1.2)	0.5 (0.2–1.1)
Correctional care during recent incarceration	---	0.4 (0.2–0.8)*	0.3 (0.1–0.6)*	---	1.2 (0.5–2.7)	1.2 (0.5–2.7)
Total days incarcerated (log)	---	1.0 (0.6–1.7)	1.0 (0.5–1.7)	---	0.8 (0.5–1.3)	0.7 (0.4–1.2)
Racial/ethnic discrimination	---	--	2.5 (1.1–5.8)*	---	---	1.2 (0.5–2.7)

*AOR = Adjusted odds ratio; CI = Confidence interval.*

*p < .05.

*Model 2: Logistic regression models adjusted for age, race/ethnicity, insurance status, total time incarcerated (log) and correctional healthcare contact during recent incarceration.*

*Model 3: Adjusted for all Model 2 covariates plus self-reported racial/ethnic discrimination and interaction between criminal record and racial/ethnic discrimination.*

## Discussion

In this cross-sectional study of 172 recently released male prisoners, we found that 42% reported a lifetime history of criminal record discrimination by healthcare workers. To our knowledge, criminal record discrimination by healthcare workers has not previously been examined. Prior studies have reported rates of general societal discrimination based on criminal record between 34-51% (Crawford et al. 2013; Young et al. 2005). These studies were conducted among active drug users in New York City and assessed self-reported discrimination based on criminal record in any setting. Our findings support and extend this work by focusing on self-reported discrimination based on criminal record experienced during interactions with doctors, nurses, psychiatrists and other healthcare workers.

Our study adds to a growing field of research on discrimination in healthcare. To date, much of this work has examined discrimination attributed to race/ethnicity (Krieger 1999). Across these studies, Blacks and Latinos more frequently report race/ethnicity-based discrimination as was the case in our sample (*data not shown*) (Shavers et al. 2012). Discrimination based on criminal record demonstrated a different pattern in our sample, however, as more than half of White participants reported criminal record discrimination compared to 41% of minority participants. Also, individuals who reported a history of healthcare discrimination based on criminal record were more likely to have obtained some post-secondary education.

Both findings may reflect the increased salience of the experience of discrimination among these relatively advantaged groups. These groups may also have experienced discrimination for the first time due to incarceration. Alternatively, less advantaged groups may be less likely to report discrimination because it is a common event in their lives. Groups with lower social statuses, such as racial minorities or those with lower education levels, often experience discrimination prior to incarceration and higher levels of discrimination overall compared with higher social status groups (Williams & Mohammed 2009). Further, among groups with multiple stigmatized identities (e.g., a high school dropout and an ex-prisoner), there may be some uncertainty as to which stigmatized characteristic to attribute an experience with discrimination (Major & O’Brien 2005). These multiple stigmatized identities may have an additive, negative effect on healthcare experiences as has been demonstrated in studies examining experiences in the job market (Pager 2003; Pager et al. 2009a) though we did not find evidence of a significant interaction between criminal record and racial/ethnic discrimination in this sample.

Individuals who reported a history of criminal record discrimination by healthcare workers were older and had more extensive incarceration histories. This finding may reflect greater time spent incarcerated and therefore at risk of discrimination associated with incarceration. Alternatively, unmeasured factors associated with length and frequency of prior incarceration may also be associated with self-reported discrimination. For example, more serious criminal offenses result in longer sentences and may be associated with differential or "unfair" treatment by healthcare workers. Longitudinal linkages between more granular criminal justice and health-related data, though challenging, are essential both for testing such a hypothesis as well as for clinical quality improvement (Matejkowski et al. 2012).

In this examination of the relationship between criminal record discrimination by healthcare workers and healthcare utilization, we found an association between self-reported criminal record discrimination by healthcare workers and increased ED utilization after adjustment for clinically relevant covariates and self-reported racial/ethnic discrimination. This finding has several potential explanations. The ED is often the initial point of contact for incarcerated individuals who need to be transferred outside the correctional system to receive healthcare. Providers caring for a patient in an orange jumpsuit and shackles likely bring biases, whether explicit or implicit, to these encounters. Implicit, or unconscious, bias based on race/ethnicity has been shown to negatively impact patient-provider interactions and deserves further study in the context of the care of incarcerated patients (Cooper et al. 2012). Alternatively, ex-prisoners in the community who perceive discrimination in healthcare settings may choose to utilize the ED more frequently given the episodic, relatively anonymous nature of these interactions. In general, while disparities in emergency care have been documented (Pletcher et al. 2008), patient-reported discrimination of all kinds in emergency settings requires further study.

Similar to prior studies, we also found a significant association between discrimination based on race/ethnicity and infrequent primary care utilization. Prior work has shown that self-reported racial/ethnic discrimination is associated with decreased adherence to recommended care, decreased utilization of preventive services and barriers to patient-provider communication (Benjamins 2012; Blanchard & Lurie 2004; Casagrande et al. 2007; Hausmann et al. 2011; Hausmann et al. 2008; Trivedi & Ayanian 2006; Van Houtven et al. 2005) though these studies have not included former prisoners. Contrary to our hypothesis, we did not find an association between criminal record discrimination by healthcare workers and infrequent primary care utilization. We believe the impact of criminal record discrimination on the patient-provider relationship warrants further study given its potential to both mediate the significant health risks following release from prison as well as to provide a point of intervention to improve health outcomes for this vulnerable group.

The results of this study should be considered in light of its limitations. First, study data are cross-sectional and therefore do not allow inferences of causation. Though we hypothesized that experiences of criminal record discrimination affect healthcare utilization patterns, we cannot rule out reverse causation, in which greater ED utilization increases exposure to criminal record discrimination. This latter explanation would still highlight a need to better understand the healthcare experiences of individuals in correctional custody, particularly those experiences occurring in ED settings. Second, participants were each in male-female relationships, were recently released from prison and were recruited from a single region in California and therefore may not be generalizable to other ex-prisoner populations or settings. Specifically, men in romantic relationships report less discrimination than their counterparts not in relationships (Kessler et al. 1999). However, recent release from prison may make experiences of discrimination more salient and thereby overestimate this exposure compared to individuals with more remote criminal justice involvement (Kressin et al. 2008). Of note, important attributes of our study sample such as rates of common chronic diseases and reported use of correctional healthcare are similar to those found in the prison population nationally (Wilper et al. 2009). Third, the small size of our sample raises the possibility of a Type II error. Inclusion of individuals with criminal justice involvement and assessment of criminal history in larger data collection efforts is needed (Ahalt et al. 2012). Next, the survey tool used to measure discrimination based on criminal record in our study was adapted from a validated measure previously used to assess racial/ethnic discrimination, the *General Ethnic Discrimination Scale.* The findings of this pilot study are hypothesis-generating but do highlight the need for such instruments to be validated in criminal justice populations. Finally, as validated measures of healthcare utilization were not present in our data, we used the most clinically relevant utilization outcomes available. Further study using validated measures and confirmed by medical record or claims data is needed.

## Conclusion

In conclusion, in a sample of 172 recently released male prisoners, we found that self-reported criminal record discrimination by healthcare workers was associated with increased ED utilization after adjusting for reported racial/ethnic discrimination and other potential confounders. We also identified a non-significant trend toward an association between this experience of discrimination and decreased utilization of primary care services. We believe our findings may have implications for both providers as well as for policy makers. Understanding the factors that influence the healthcare utilization of ex-prisoner populations will become increasingly relevant as health insurance expansions result in Medicaid eligibility for nearly 250,000 individuals leaving correctional facilities each year (Cuellar & Cheema 2012). More than 40% of ex-prisoners in this study reported a history of healthcare discrimination based on criminal record, and patterns of healthcare utilization varied based on participants’ experiences with discrimination. For healthcare workers and policymakers alike, greater understanding of criminal record discrimination may offer opportunities to increase patient engagement and improve access for this vulnerable population.

## Authors’ original submitted files for images

Below are the links to the authors’ original submitted files for images. Authors’ original file for figure 1

## References

[CR1] Ahalt C, Binswanger IA, Steinman M, Tulsky J, Williams BA (2012). Confined to ignorance: the absence of prisoner information from nationally representative health data sets. Journal of General Internal Medicine.

[CR2] Benatar SR, Upshur RE (2008). Dual loyalty of physicians in the military and in civilian life. American Journal of Public Health.

[CR3] Benjamins MR (2012). Race/ethnic discrimination and preventive service utilization in a sample of whites, blacks, Mexicans, and Puerto Ricans. Medical Care.

[CR4] Binswanger I, Krueger P, Steiner J (2009). Prevalence of chronic medical conditions among jail and prison inmates in the USA compared with the general population. Journal of Epidemiology & Community Health.

[CR5] Binswanger IA, Stern MF, Deyo RA, Heagerty PJ, Cheadle A, Elmore JG, Koepsell TD (2007). Release from prison–a high risk of death for former inmates. New England Journal of Medicine.

[CR6] Blanchard J, Lurie N (2004). R-E-S-P-E-C-T: patient reports of disrespect in the health care setting and its impact on care. Journal of Family Practice.

[CR7] Casagrande SS, Gary TL, LaVeist TA, Gaskin DJ, Cooper LA (2007). Perceived discrimination and adherence to medical care in a racially integrated community. Journal of General Internal Medicine.

[CR8] Cooper LA, Roter DL, Carson KA, Beach MC, Sabin JA, Greenwald AG, Inui TS (2012). The associations of clinicians’ implicit attitudes about race with medical visit communication and patient ratings of interpersonal care. American Journal of Public Health.

[CR9] Crawford ND, Ford C, Galea S, Latkin C, Jones KC, Fuller CM (2013). The relationship between perceived discrimination and high-risk social ties among illicit drug users in new york city, 2006-2009. AIDS and Behavior.

[CR10] Cuellar AE, Cheema J (2012). As roughly 700,000 prisoners are released annually, about half will gain health coverage and care under federal laws. Health Affairs (Project Hope).

[CR11] Glaze LE, Bonczar TP (2011). Probation and Parole in the United States, 2010.

[CR12] Guerino P, Harrison PM, Sabol WJ (2011). Prisoners in 2010.

[CR13] Hausmann LR, Hannon MJ, Kresevic DM, Hanusa BH, Kwoh CK, Ibrahim SA (2011). Impact of perceived discrimination in healthcare on patient-provider communication. Medical Care.

[CR14] Hausmann LR, Jeong K, Bost JE, Ibrahim SA (2008). Perceived discrimination in health care and use of preventive health services. Journal of General Internal Medicine.

[CR15] Kessler RC, Mickelson KD, Williams DR (1999). The prevalence, distribution, and mental health correlates of perceived discrimination in the United States. Journal of Health and Social Behavior.

[CR16] Klonoff EA, Landrine H (1999). Cross-validation of the schedule of racist events. Journal of Black Psychology.

[CR17] Kressin NR, Raymond KL, Manze M (2008). Perceptions of race/ethnicity-based discrimination: a review of measures and evaluation of their usefulness for the health care setting. Journal of Health Care for the Poor and Underserved.

[CR18] Krieger N (1999). Embodying inequality: a review of concepts, measures, and methods for studying health consequences of discrimination. International Journal of Health Services.

[CR19] Kulkarni SP, Baldwin S, Lightstone AS, Gelberg L, Diamant AL (2010). Is incarceration a contributor to health disparities? Access to care of formerly incarcerated adults. Journal of Community Health.

[CR20] Landrine H, Klonoff EA, Corral I, Fernandez S, Roesch S (2006). Conceptualizing and measuring ethnic discrimination in health research. Journal of Behavioral Medicine.

[CR21] LaVeist TA, Nickerson KJ, Bowie JV (2000). Attitudes about racism, medical mistrust, and satisfaction with care among African American and white cardiac patients. Medical Care Research and Review.

[CR22] Major B, O’Brien LT (2005). The social psychology of stigma. Annual Review of Psychology.

[CR23] Marlow E, White MC, Chesla CA (2010). Barriers and facilitators: parolees’ perceptions of community health care. Journal of Correctional Health Care.

[CR24] Maruschak L, Beavers R (2009). HIV in Prisons, 2007-08.

[CR25] Matejkowski J, Harron A, Festinger DS (2012). Opportunities for Information-Sharing Between Criminal Justice And Community Substance Abuse Treatment Systems: Community Oriented Correctional Health Services.

[CR26] Mellow J, Greifinger RB (2007). Successful reentry: the perspective of private correctional health care providers. Journal of Urban Health.

[CR27] Minton TD (2011). Jail Inmates at Midyear 2010 - Statistical Tables.

[CR28] Newman WJ, Scott CL (2012). Brown v. Plata: Prison Overcrowding in California. J Am Acad Psychiatry Law.

[CR29] Pager D (2003). The mark of a criminal record. American Journal of Sociology.

[CR30] Pager D, Shepherd H (2008). The sociology of discrimination: racial discrimination in employment, housing, credit, and consumer markets. Annual Review of Sociology.

[CR31] Pager D, Western B, Bonikowski B (2009). Discrimination in a low-wage labor market: a field experiment. American Sociological Review.

[CR32] Pager D, Western B, Sugie N (2009). Sequencing disadvantage: barriers to employment facing young black and white men with criminal records. The Annals of the American Academy of Political and Social Science.

[CR33] Paradies Y (2006). A systematic review of empirical research on self-reported racism and health. International Journal of Epidemiology.

[CR34] Patterson EJ (2013). The dose-response of time served in prison on mortality: New York State, 1989-2003. American Journal of Public Health.

[CR35] Pletcher MJ, Kertesz SG, Kohn MA, Gonzales R (2008). Trends in opioid prescribing by race/ethnicity for patients seeking care in US emergency departments. JAMA.

[CR36] Schnittker J, John A (2007). Enduring stigma: the long-term effects of incarceration on health. Journal of Health and Social Behavior.

[CR37] Shavers VL, Fagan P, Jones D, Klein WM, Boyington J, Moten C, Rorie E (2012). The state of research on racial/ethnic discrimination in the receipt of health care. American Journal of Public Health.

[CR38] Smedley BD, Stith AY, Nelson AR (2003). Unequal Treatment: Confronting Racial and Ethnic Disparities in Health Care.

[CR39] Pew Center on the States (2009). One in 31: The Long Reach of American Corrections.

[CR40] Trivedi AN, Ayanian JZ (2006). Perceived discrimination and use of preventive health services. Journal of General Internal Medicine.

[CR41] Uggen C, Manza J (2002). Democratic contraction? Political consequences of felon disenfranchisement in the United States. American Sociological Review.

[CR42] Uggen C, Manza J, Behrens A (2004). Less than the Average Citizen: Stigma, Role Transition, and the Civic Reintegration of Convicted Felons.

[CR43] Van Houtven CH, Voils CI, Oddone EZ, Weinfurt KP, Friedman JY, Schulman KA, Bosworth HB (2005). Perceived discrimination and reported delay of pharmacy prescriptions and medical tests. Journal of General Internal Medicine.

[CR44] Wang EA, Wang Y, Krumholz HM (2013). A high risk of hospitalization following release from correctional facilities in Medicare beneficiaries: a retrospective matched cohort study, 2002 to 2010. JAMA Internal Medicine.

[CR45] Williams DR, Mohammed SA (2009). Discrimination and racial disparities in health: evidence and needed research. Journal of Behavioral Medicine.

[CR46] Wilper AP, Woolhandler S, Boyd JW, Lasser KE, McCormick D, Bor DH, Himmelstein DU (2009). The health and health care of US prisoners: results of a nationwide survey. American Journal of Public Health.

[CR47] Young M, Stuber J, Ahern J, Galea S (2005). Interpersonal discrimination and the health of illicit drug users. American Journal of Drug and Alcohol Abuse.

