# Supplementary figures and images for: Discrimination based on criminal record and healthcare utilization among men recently released from prison: a descriptive study
Source: Health Justice. 2014 Mar 25;2:6. doi: 10.1186/2194-7899-2-6 (PMC4308970; doi:10.1186/2194-7899-2-6)

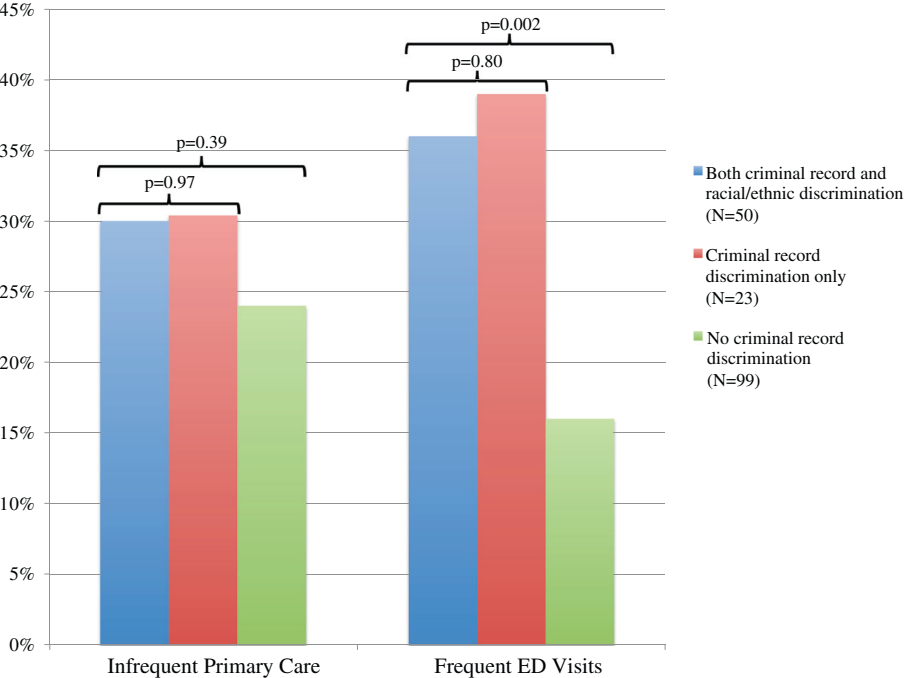

Supplement: Supplementary file 1 — Authors’ original file for figure 1 [file 40352_2013_10_MOESM1_ESM.pdf]
